# Shedding dynamics of Morogoro virus, an African arenavirus closely related to Lassa virus, in its natural reservoir host *Mastomys natalensis*

**DOI:** 10.1038/srep10445

**Published:** 2015-05-29

**Authors:** Benny Borremans, Raphaël Vossen, Beate Becker-Ziaja, Sophie Gryseels, Nelika Hughes, Mats Van Gestel, Natalie Van Houtte, Stephan Günther, Herwig Leirs

**Affiliations:** 1 Evolutionary Ecology Group, University of Antwerp, Antwerp, Belgium; 2 Bernhard-Nocht-Institute for Tropical Medicine, Hamburg, Germany

## Abstract

Arenaviruses can cause mild to severe hemorrhagic fevers. Humans mainly get infected through contact with infected rodents or their excretions, yet little is known about transmission dynamics within rodent populations. Morogoro virus (MORV) is an Old World arenavirus closely related to Lassa virus with which it shares the same host species *Mastomys natalensis*. We injected MORV in its host, and sampled blood and excretions at frequent intervals. Infection in adults was acute; viral RNA disappeared from blood after 18 days post infection (dpi) and from excretions after 39 dpi. Antibodies were present from 7 dpi and never disappeared. Neonatally infected animals acquired a chronic infection with RNA and antibodies in blood for at least 3 months. The quantified excretion and antibody patterns can be used to inform mathematical transmission models, and are essential for understanding and controlling transmission in the natural rodent host populations.

Several viruses of the Arenaviridae family cause mild to severe disease in humans across the world. They belong to a group of viruses that cause often-lethal hemorrhagic fevers, which also includes members of the Bunyaviridae, Filoviridae and Flaviviridae^1^,^2^. All known arenaviruses are zoonotic, and most are carried by rodents, with each virus having its own specific natural reservoir host species^3^. Humans can get infected through close contact with infected rodents or their excretions, and human-to-human transmission is rare except in some nosocomial situations. It is therefore crucial to understand arenavirus transmission dynamics within rodent host populations^4^.

For rodent-borne zoonotic diseases, the force of infection to humans largely depends on the intensity of rodent-human contact and on the duration, mode and quantity of virus shedding by the rodent^5^. In the case of arenaviruses, information about shedding dynamics is sparse, and it is known that not all arenaviruses share the same infection pattern. Some, such as Whitewater Arroyo virus and Catarina virus, seem to induce a transient acute infection and lifelong immunity, while others establish a chronic infection^6^,^7^,^8^,^9^. Although knowledge of excretion patterns is an essential basis for understanding transmission dynamics, there is a dearth of information due to low time resolutions of the few existing experimental infection studies.

In this study, we present the results of an experimental infection of the East-African Old World arenavirus Morogoro virus (MORV) in its natural rodent host, the Natal Multimammate Mouse *Mastomys natalensis*. MORV is an outlier strain of Mopeia virus, and shares very similar characteristics. Importantly, it is closely related to Lassa virus (LASV) and even has the same rodent host species^10^. LASV only occurs in West-Africa and causes hemorrhagic Lassa fever^11^, while MORV occurs in Tanzania and does not seem to be pathogenic to humans^12^. As Mopeia virus and MORV share some important characteristics with LASV, both have been used successfully as safe surrogate models for LASV in studies on vaccine development^13^,^14^, pathogenesis^15^,^16^, immunology^17^,^18^ and ecology^19^. We aimed to characterize the infection and virus excretion patterns of MORV in its natural rodent host under laboratory conditions. Blood and excretions were sampled at regular intervals for the duration of the rodent’s natural lifetime, and antibody and MORV RNA levels were measured.

## Methods

### Animal experiments

*M. natalensis* individuals were taken from a breeding colony established in 1999 from arenavirus-free individuals wild-caught in Morogoro (Tanzania). During the experiment, animals were housed individually in polyester ventilated HEPA-filter equipped microisolator cages (ISOcage^®^, Tecniplast Italy) to prevent contamination of the environment and other individuals. All manipulations of infectious materials and experimental animals were performed under biosafety level 2+ conditions. Microisolator cages were only opened under a laminar flow biosafety cabinet (type II) to prevent environmental or experimenter contamination. Virkon^®^ (Antec International) was used for decontamination of potentially infected surfaces and materials. Animals were checked daily for signs of illness or stress, and food and water were provided ad libitum. Prior to inoculation, none of the animals was infected with MORV. Animals were assigned randomly to a control or treatment group with equal sex ratios and age distributions in each group. Body weights did not differ significantly between the two groups (t = 0.61, df = 13, p = 0.56).

### Virus and inoculation

Animals were inoculated with MORV isolated from *M. natalensis* serum, strain 3017/2004 passaged 4 times in Vero cells^10^. This strain originated from a *M. natalensis* individual caught in Morogoro (Tanzania) in 2004, and thus has the same geographic origin as the experimental animals used in this study. Fifteen mice (aged between 55 and 130 days) were inoculated intra-peritoneally with 1 × 10^4^ focus forming units (FFU) in 0.1 mL phosphate buffered saline (PBS), pH 7.5. Eight control mice were inoculated intra-peritoneally with 0.1 mL PBS.

### Sampling

Blood and excretion samples were taken at regular intervals organized so that a high resolution of days post-infection (p.i.) was obtained. For sampling, animals were transferred to a transparent polyester zip lock seal bag until spontaneous urination or up to 15 minutes. Urine was collected by pipetting from the plastic bag or directly from the animal. Saliva was sampled by pipetting directly from the mouth while holding the mouse by the scruff of the neck. After saliva pipetting, a small piece of filter paper (Serobuvard^®^, LDA^22^ Zoopole, France) was put in the mouth for a few seconds to collect additional saliva. Feces were collected after spontaneous defecation. After excretion sampling the mice were returned to the plastic bag and anesthetized by adding isoflurane (IsoFlo^®^ Abbott Logistics B.V., Breda, Netherlands) on cotton wool. While mice were sedated, a blood sample was taken from the retro-orbital plexus using a 70 μL hematocrit capillary. Thirty μL of blood was transferred to filter paper, while the rest was centrifuged at 800 × *g* for 1 min to separate serum. Immediately after sampling, all samples (excl. filter paper samples) were transferred to liquid nitrogen for the duration of the sampling session, after which they were stored at –80 °C. Filter paper samples were stored at -20 ° C in an envelope in a zip-lock sealed bag containing silica for low humidity. Mice were weighed during sampling sessions using a spring balance with 1 g precision. Body mass index was initially planned to be used as a measure of condition, but was eventually not used because the error on length measurements was too large. After the last sampling session (210–211 days p.i.), animals were sacrificed with an isoflurane overdose followed by cervical dislocation. All animal work was done according to relevant EU guidelines and approved by the University of Antwerp Ethical Committee for Animals (2012–03).

### Intranasal inoculation of excretions

To test whether excretions that tested positive for MORV RNA can indeed infect susceptible mice, we inoculated 10 mice intranasally with a homogenate of positive urine (5 mice) or feces (5 mice) samples. Each homogenate consisted of 10 μL from each of 6 samples that tested positive for MORV RNA, and was pipetted in and on the nostrils. It was not possible to test whether RNA-positive saliva was infectious because the volumes were too small for both MORV RT-PCR and inoculation.

### Inoculation of newborns

To test whether a chronic infection would develop when infection occurs at a very young age, we inoculated a litter of 5 neonatal *M. natalensis* (1-day old). Neonates were inoculated intranasally with a lower dose than adults (10 μL of 10^4^ FFU/mL). On days 24, 32, 38, 49, 54, 66 and 87 after infection, blood and urine samples were taken and analysed for presence of MORV RNA and anti-MORV antibodies.

### Extraction of RNA

Extraction of viral RNA for quantitative MORV RT-PCR was done using the QIAmp Viral RNA Mini Kit (Qiagen, Hilden, Germany), with adapted lysis protocols for different sample types. For lysis from urine and saliva, the maximum possible amount of sample up to a volume of 10 μL was pipetted and transferred to 150 μL AVL buffer (incl. 1.5 μL carrier RNA). Following the protocol of Hardestam *et al.*^20^, weighed feces (±20 mg) was dissolved in 600 mL PBS, centrifuged at 1,800 x *g* for 5 min, and 140 μL supernatant was transferred to 560 μL AVL-carrier RNA buffer. Samples in AVL were stored at –20 °C until continuing the extraction protocol. We tested whether freezing of samples in AVL would affect quantitative RT-PCR results: this was not the case. From this point, manufacturer instructions were followed, with a final elution in 25 μL RNase-free water that was stored at –20 °C until down-stream procedures within 2 weeks time. When saliva could not be sampled by pipetting, saliva on filter paper was used as a source for RNA extraction and RT-PCR, following the protocol described above. A quantity of 5 μL saliva was estimated to be on the filter paper and was used for quantification. Whole blood was extracted from samples stored frozen in capillaries when available, but when it was not possible to sample enough blood for both filter paper and capillary storage, filter paper was used as the primary storage medium. One filter paper sample (a calibrated pre-punched circle) contained 10 μL of whole blood. Extraction of whole blood was done as described above, using a starting AVL quantity of 300 μL.

### Real-time RT-PCR

We performed one-step TaqMan^®^ real-time RT-PCR’s to evaluate the relative quantity of MORV in the RNA extracts while co-amplifying beta-actin mRNA (host housekeeping gene) to assess RNA extract quality. The latter was only applicable to RNA extracts of whole blood samples dried on filter paper, and not to cell-free excreta or serum samples. To keep consistency across all samples the same multiplex protocol was used for all sample types. MORV primers (Moro-F 5′-TCCTATGTTGATGCTGCAAACC-3′ and Moro-R 5′-ACCAGTACATCCTCCTAAAGGTATCC-3′) and MGB-Probe (Moro-P 5′- 6-FAM-TGTGGAACCATCGCC-MGBNFQ-3′) were designed to target a portion of the GPC gene. β-actine primers (BAct-F 5′-CCGTAAAGACCTCTATGCCAACAC-3′ and BAct-R 5′-CGCTCAGGAGGAGCAATGA-3′) and probe (BAct-P 5′-VIC-CACCATGAAGATCAAG-MGBNFQ-3′) were designed across two exomes of the rodent β-actine gene based on *Mus musculus* and *Rattus norvegicus* sequences. In a total volume of 10 μL per reaction, 1 x TaqMan^®^ Fast Virus 1-Step Master Mix (Life Technologies) was mixed with 0.4 μM BAct-F, 0.9 μM BAct-R, 0.25 μM BAct-P, 0.6 μM Moro-F, 0.6 μM Moro-R, 0.3 μM Moro-P, 2.45 μL RNase-free water and 2 μL of RNA template. One step RT-PCR’s were run on a StepOnePlus Lightcycler (Life Technologies), with 5 minutes at 50 °C, 20 seconds at 95 °C, and 40 cycles of 3 seconds at 95 °C followed by 30 seconds at 60 °C. All samples were run in quantitative RT-PCR reactions using duplicate serial dilutions of a RNA standard extracted from MORV cell culture supernatant (10^6^ FFU/mL) to allow relative quantitation. Relative RNA quantities in specimens were adjusted according to the used sample weight or volume to obtain relative copy numbers per gram or mL, respectively. Because sample volumes were often too low (e.g. 0.5–2 μL for saliva) for the use of plaque assays for virus quantification, we used viral RNA as a proxy for infectious virus. The presence of viral RNA can derive from infectious as well as non-infectious particles, but while the exact proportion of non-infectious/infectious virus is unknown, many studies have observed a close correlation between the amount of viral RNA and infectious virus^20^,^21^,^22^,^23^,^24^,^25^,^26^.

We tested whether RT-PCR was inhibited by the sample. This was done for blood and excretions by comparing the intercept of a standard curve of control samples (10^6^ FFU/mL; dilutions 10^−4^,10^−5^,10^−6^) with the intercept of the same standard curve to which 1 μL of negative RNA extract was added.

### Antibody quantification

The presence of anti-MORV IgG antibodies was determined by immuno-fluorescence assay^9^. Antibody titers were estimated using dilution series, the exact dilutions for which were chosen by narrowing down from initial rough 20-fold dilution steps to limit the number of dilutions needed for a sufficiently low estimation error. Dried blood spots that did not elute in PBS were eluted after addition of NH_3_ as described in^27^, but although this method improved elution, it was still not complete. To account for the lower antibody titers of fixed samples due to imperfect blood elution, the spectrophotometric absorption values after elution in PBS were used to estimate the correct titer value by normalizing against a standard curve of absorption values of perfectly eluted samples.

### Statistics and data preparation

Differences between age-normalized growth rates were tested using a Welch Two Sample t-test. Sex ratio differences were tested using an Exact Binomial Test and a permutation test (1,000,000 iterations) without replacement. Age effects were tested using a Chi-squared test of a generalized linear model with logit link function. Data entry was done using Excel (Microsoft). Data preparation, statistics and plotting was done in R Statistical Software^28^, Means are always shown with standard errors.

## Results

### Inhibition

There was RT-PCR inhibition (i.e. decreased RT-PCR efficiency due to inhibiting particles that may be present in the sample) of MORV RNA in whole blood, resulting in a fairly strong yield reduction, but excretions did not seem to inhibit RT-PCR (Figure S1). As inhibition resulted in quantities that were three times lower than the control samples, estimated MORV RNA quantities were multiplied by 3 in all figures showing RNA in blood.

### Neonatal infection

All five inoculated neonates got infected; for all tested days (between 24 and 87 dpi), urine samples tested positive for MORV RNA and blood samples tested positive for MORV RNA and antibodies.

### Weight

**Figure 1:**
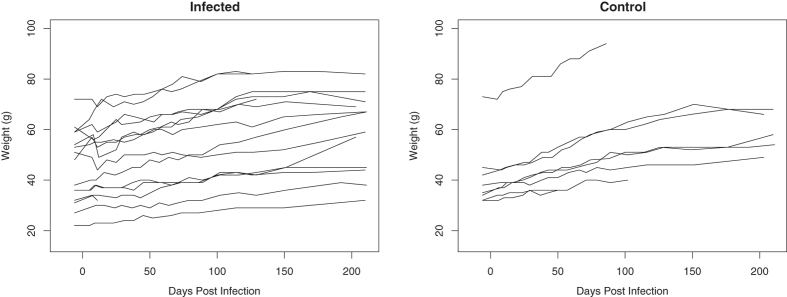
Body weight vs. days post-infection for infected and control mice.

Overall, age-corrected growth rates were not significantly affected by infection status (mean rates 0.08 ± 0.01 g/day (infected) vs. 0.12 ± 0.02 g/day (control); t = 1.5, df = 10.7, P = 0.18). Between 7 and 15 days p.i. a drop in body weight was observed for 6 out of 15 infected animals (Fig. 1). The mean weight decrease was 7 ± 0.8% of the body weight the animal should have had at that time according to the estimated growth curve. All of these animals recovered from this weight loss, and had a normal growth rate (estimated for the entire duration of the experiment) compared to control animals (t = 0.13, df = 12.1, P = 0.9). The six animals that experienced weight loss were all male. When tested using a binomial test and considering the sex ratio in the group of animals that were inoculated successfully, this sex effect was not significant (P = 0.19, Exact Binomial test). The presence of weight loss was not correlated with age (*χ*_1,13_ = 0.05, P = 0.83), but was significantly correlated with body weight at the time of infection, where those that experienced weight loss were heavier than those that did not (54.3 ± 5.5 g vs. 39.2 ± 4.2 g; *χ*_1,13_ = 4.55, P = 0.03).

### Blood

**Figure 2:**
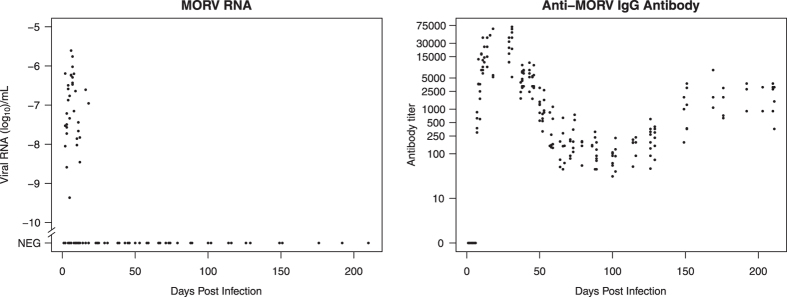
Anti-MORV IgG antibody and MORV RNA levels in blood. Note the different Y-axis limits. Relative RNA levels are log-transformed (log_10_ [RNA level]) to improve plot interpretation. A value of -6 corresponds with the RNA level of a 10^−6^ dilution of the standard RNA (used for relative quantification) extracted from a 10^6^ FFU/mL virus stock, and would thus correspond with a concentration of 1 FFU/mL. RNA results originating from blood stored on filter paper and in capillaries were pooled for plotting purposes.

Antibodies were not detected until day 7 p.i., but were present on or right after day 7 p.i. for all infected animals (Fig. 2). Antibody levels increased sharply to high titers (>50,000), peaking around day 20 p.i., followed by a decrease phase until approx. day 70 p.i. during which titers reach minimum levels of approx. 100. A secondary slower increase then started around day 120 p.i., with antibody titers reaching a final equilibrium level (±1,000) from approx. day 160 p.i.. MORV RNA was detectable in blood from day 2 p.i., levels increased until day 7–8 p.i., and sharply declined until disappearing completely from approx. day 12–18 p.i. onwards (Fig. 2). This pattern was observed for all animals. Individual plots of antibody and RNA dynamics in blood are shown in the Supplementary Information (Figures S2 to S16).

### Excretions

Patterns of viral RNA presence and quantity were similar between urine, saliva and feces (Fig. 3). Viral RNA was detectable in feces from day 3 p.i. until day 29 p.i., in saliva from day 3 p.i. until day 39 p.i., and in urine from day 6 to 29 p.i.. The peak of viral shedding occurred around day 10 p.i. in all excretions. There was considerable individual variation in shedding dynamics, but without a clear pattern. Individual plots of antibody and RNA dynamics in excretions are shown in the Supplementary Information (Figures S2 to S16).

Out of 10 mice that were intranasally inoculated with pooled RNA-positive excretions, 6 seroconverted, showing that MORV RNA in excretions indeed represents infectious virus.

## Discussion

Infection with MORV seems to induce minor transient disease in its natural host *M. natalensis*. In 40% of infected mice, a body weight loss of about 7% was observed around 10 days after infection, but this was temporary and growth rates appeared to be unaffected. This mild effect is similar to that observed for LASV in *M. natalensis*, where no signs of clinical disease were observed^29^, but contrasts with patterns seen in other arenaviruses. For example, Junin virus infection in newborn *Calomys musculinus* can result in severely decreased survival probability^30^. Similarly, Machupo virus infection has been observed to cause adverse effects in its natural host *Calomys callosus*^31^, and Lymphocytic Choriomeningitis virus (LCMV) is known to severely affect its host, depending on the age at the time of infection^32^.

We observed that animals with a higher body weight (but not age) at the time of infection were more likely to experience temporary weight loss. The reason for this is not clear, but one explanation may be that a better body condition correlates with a stronger immune response that requires more body resources, resulting in temporary weight loss^33^.

The persistence of antibodies until the last moment of sampling, combined with the disappearance of viral RNA from blood or excretions, suggests that adult *M. natalensis* develop lifelong immunity against MORV. Although we did not test whether these animals would be immune to re-infection, the fact that animals in natural conditions are very rarely positive for both viral RNA and antibodies supports the hypothesis of lifelong immunity^19^. A similar pattern that suggests lifelong immunity has also been observed for Whitewater Arroyo virus in *Neotoma albigula*, Catarina virus in *Neotoma micropus* and Tamiami virus in *Sigmodon hispidus*^5^,^34^,^35^. Lifelong immunity has been observed for LASV in *M. natalensis*, but only when infected as adults. As for LASV^29^, the animals infected as neonates in our study became chronically infected with simultaneous development of antibodies.

For MORV as well as for LASV, the simultaneous presence of RNA and antibodies in experimental laboratory animals contrasts with the patterns seen in nature, where only a small percentage of animals are positive for both^19^,^36^. Plausible explanations for this are passive immunity through maternal antibodies^37^, and the fact that newborns remain in their mother’s burrow for about 3 weeks^38^, which would render it unlikely that newborns from a non-infected mother come into contact with the virus. This then implies that the existence of chronically infected individuals in natural conditions is rare, and perhaps negligible for population-scale transmission dynamics. A contrasting hypothesis would be that newborns can get infected and antibodies wane after some time. Although this hypothesis better fits field data, we did not see any evidence of antibody waning, as neonatally infected animals remained antibody and RNA positive for at least 3 months post inoculation. Nevertheless it must be noted that in this study only 1 family of newborns was used, so although chronic infection is clearly possible in laboratory conditions these results are not entirely conclusive, and a more detailed experiment in laboratory and field conditions should be conducted.

The temporal antibody dynamics of MORV infection, with antibody titers reaching a first peak soon after infection, and a second increase around 120–140 days p.i., are similar to those seen in LCMV in Syrian hamsters^39^. This antibody pattern was the same for all animals, and in fact showed remarkably little variation in timing and titer (Fig. 2 and Supplementary Information Figures S2-S16). We do not know whether this pattern would appear in natural conditions or when using a different dose or inoculation route, which are all factors known to affect the course of an infection^40^. Here, there is again some contrast with the immune response towards New World arenaviruses. Adult *C. musculinus*, when infected with Junin virus, become immune in only 50% of cases, with the other half developing a chronic infection without antibody response^41^. Chronic infection without immunity is seen for Machupo virus in *C. callosus*, for Guanarito virus in newborn and juvenile *Zygodontomys brevicauda*, and for LCMV in white mice^31^,^42^,^43^. Adult *Z. brevicauda* seem to acquire a transient immunity against Guanarito virus while developing a chronic infection.

Unexpectedly, we observed a secondary rise in antibody titer to an equilibrium around day 160 p.i.. This rise has also been observed for LCMV in Syrian hamsters^39^. A plausible explanation for this is that two types of IgG antibodies are detected using the assay in our study, and that the secondary rise to an equilibrium level is actually the detection of a more slowly maturing type of IgG antibody^44^.

The different immune responses of arenaviruses appear to be correlated with the shedding patterns. In animals infected as adults, we observed a short viraemia and a transient shedding period, followed by clearance of MORV RNA and lifelong presence of antibodies. While we did not test organs for MORV RNA presence, we never detected any sign of virus presence in blood or excretions after initial clearance, suggesting that there was no chronic or latent infection. In contrast, all neonatally infected animals developed a chronic infection with antibody development. This age-at-infection effect has also been observed for LASV, Tamiami virus, Catarina virus and Whitewater Arroyo virus^6^,^29^,^34^,^35^, but seems to be absent for other arenaviruses, where infection is always chronic without antibody development^31^,^32^,^41^,^42^,^43^.

This fundamental difference between infectivity patterns is likely to have important consequences for enzootic transmission dynamics. When rodent demography is known, knowledge about whether the virus belongs to the former (chronic + acute) or latter (always chronic) group can inform mathematical transmission models, and can thus be used to understand and predict changes in the force of infection to humans.

## Additional Information

**How to cite this article**: Borremans, B. *et al.* Shedding dynamics of Morogoro virus, an African arenavirus closely related to Lassa virus, in its natural reservoir host *Mastomys natalensis. Sci. Rep.* **5**, 10445; doi: 10.1038/srep10445 (2015).

## Supplementary Material

Supplementary Information

## Figures and Tables

**Figure 3:**
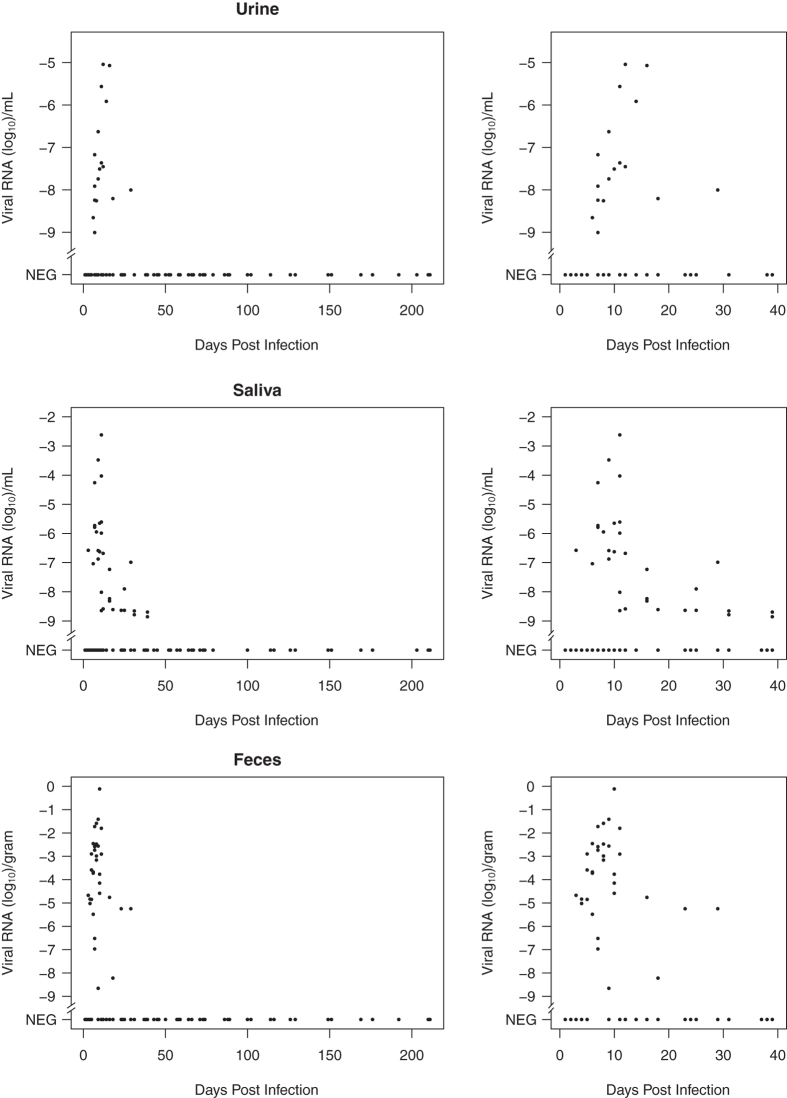
Relative MORV RNA quantities in urine, saliva and feces, for the entire duration of the experiment, and for the shedding period at higher resolution. Relative RNA levels are log-transformed (log_10_ [RNA level]) to improve plot interpretation. A value of -6 corresponds with the RNA level of a 10^−6^ dilution of the standard RNA (used for relative quantification) extracted from a 10^6^ FFU/mL virus stock, and would thus correspond with a concentration of 1 FFU/mL.
