# Supplementary material for: Shedding dynamics of Morogoro virus, an African arenavirus closely related to Lassa virus, in its natural reservoir host Mastomys natalensis
Source: Sci Rep. 2015 May 29;5:10445. doi: 10.1038/srep10445 (PMC4448520; doi:10.1038/srep10445)
Supplement: Supplementary Information [file srep10445-s1.pdf]

Supplementary Information:

Shedding dynamics of Morogoro virus, an African arenavirus closely related to Lassa virus, in its natural reservoir host *Mastomys natalensis*

Benny Borremans[1], Raphaël Vossen[1], Beate Becker-Ziaja[2], Sophie Gryseels[1], Nelika Hughes[1], Mats Van Gestel[1], Natalie Van Houtte[1], Stephan Günther[2], Herwig Leirs[1]

[1]Evolutionary Ecology Group, University of Antwerp, Antwerp, Belgium

[2]Bernhard-Nocht-Institute for Tropical Medicine, Hamburg, Germany

Corresponding author: B.B. (benny.borremans@uantwerpen.be)

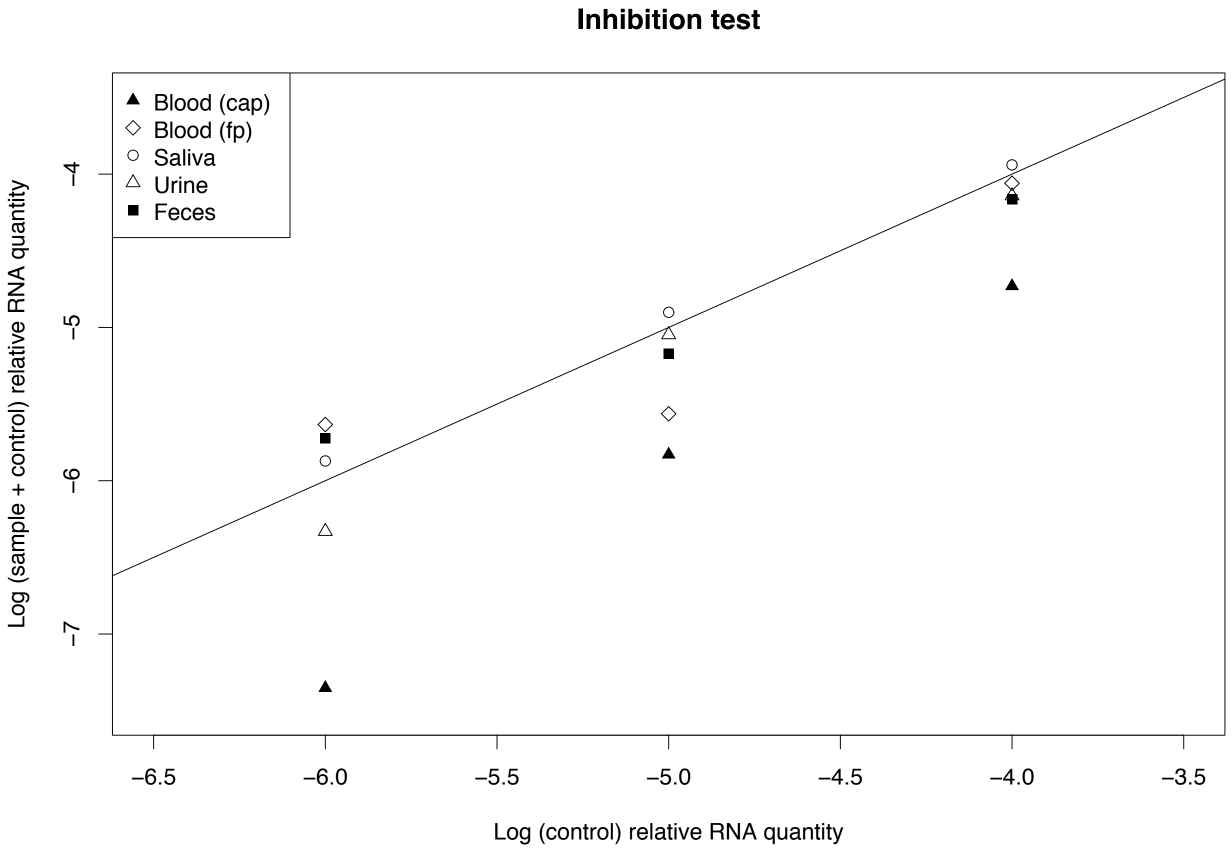

Figure 1. Inhibition test results for blood from capillaries (cap) and filter paper (fp), saliva, urine and feces. The line shows the estimated standard curve as a reference.

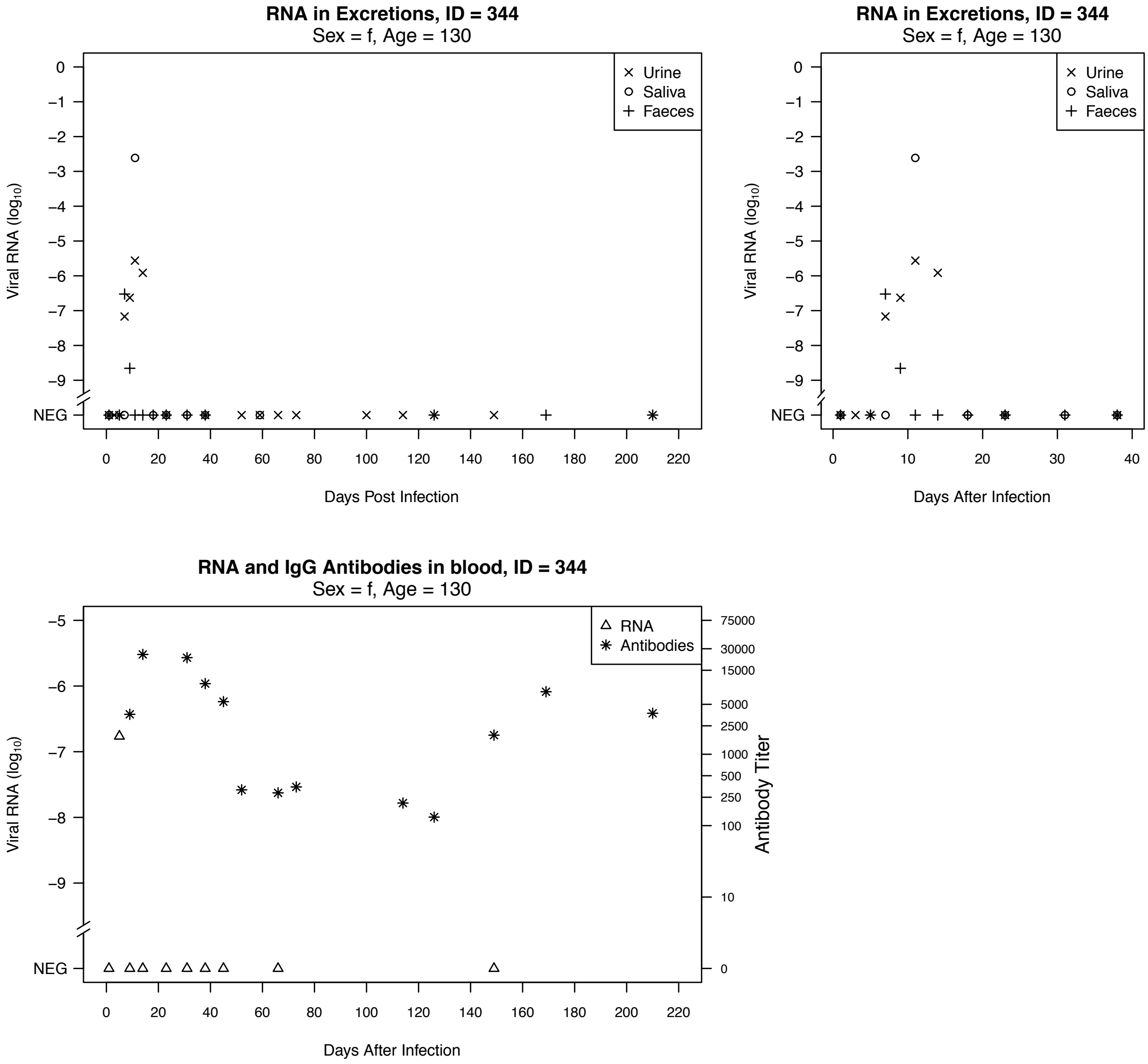

Figure 2. Antibody titers (blood) and MORV RNA (blood, excretions) for individual 1 of 15. Relative RNA levels are log-transformed ($\log_{10}$ [RNA level]) to improve plot interpretation. A value of -6 corresponds with the RNA level of a 10^{-6} dilution of the standard RNA (used for relative quantification) extracted from a 10^{6} FFU/mL virus stock, and would thus correspond with a concentration of 1 FFU/mL.

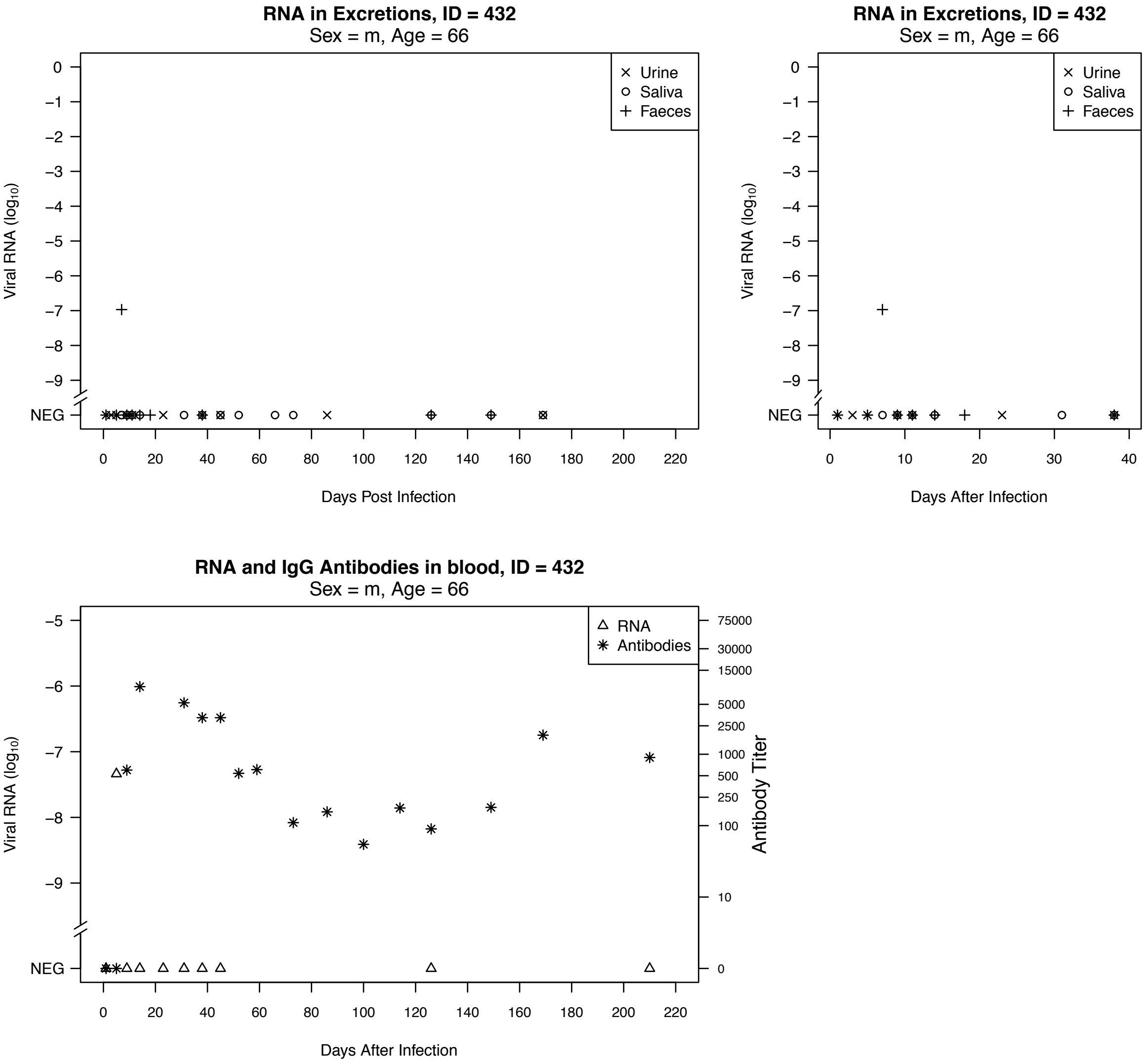

Figure 3. Antibody titers (blood) and MORV RNA (blood, excretions) for individual 2 of 15. Relative RNA levels are log-transformed ($\log_{10}$ [RNA level]) to improve plot interpretation. A value of -6 corresponds with the RNA level of a 10^{-6} dilution of the standard RNA (used for relative quantification) extracted from a 10^{6} FFU/mL virus stock, and would thus correspond with a concentration of 1 FFU/mL.

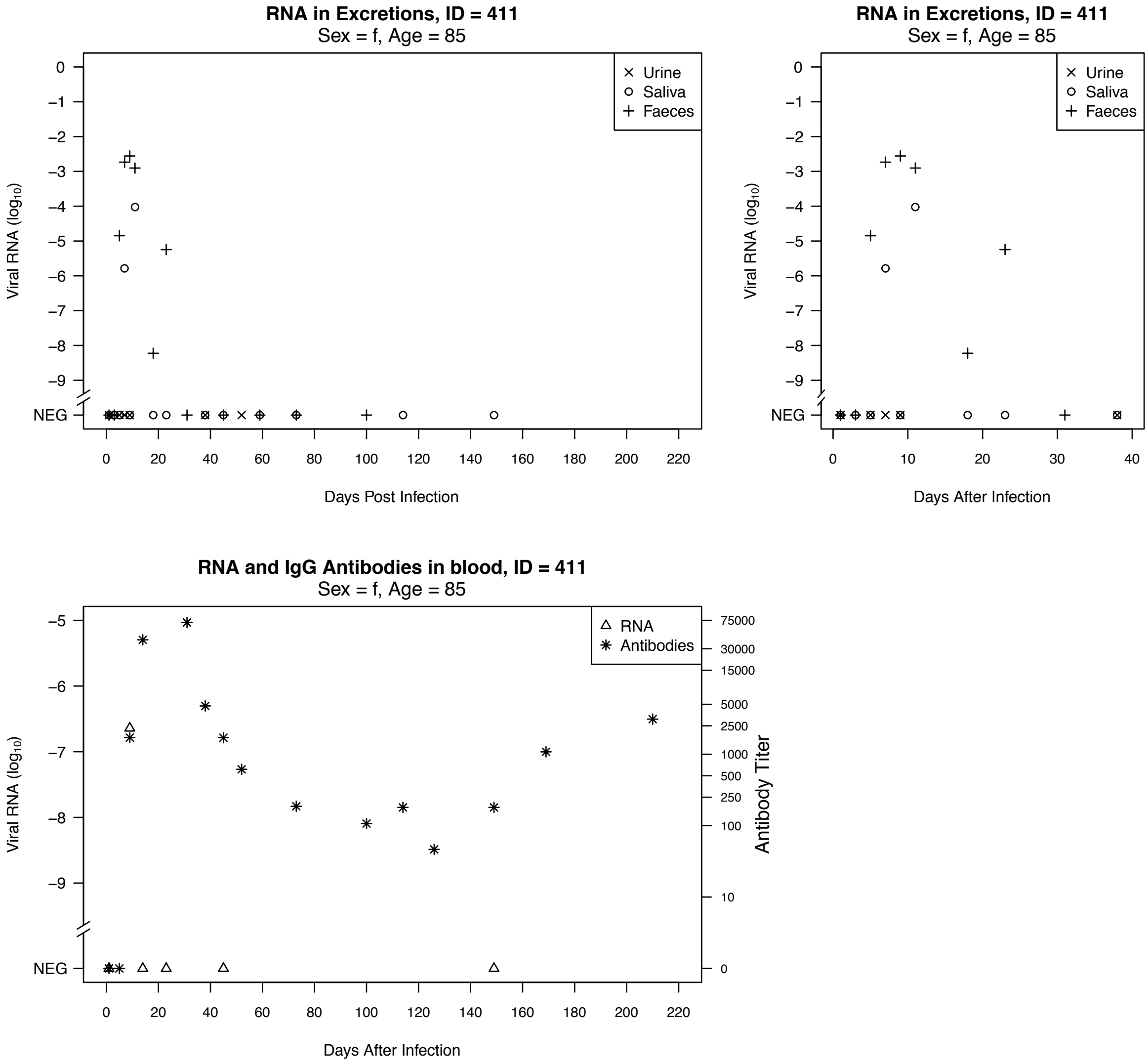

Figure 4. Antibody titers (blood) and MORV RNA (blood, excretions) for individual 3 of 15. Relative RNA levels are log-transformed ($\log_{10}$ [RNA level]) to improve plot interpretation. A value of -6 corresponds with the RNA level of a 10^{-6} dilution of the standard RNA (used for relative quantification) extracted from a 10^{6} FFU/mL virus stock, and would thus correspond with a concentration of 1 FFU/mL.

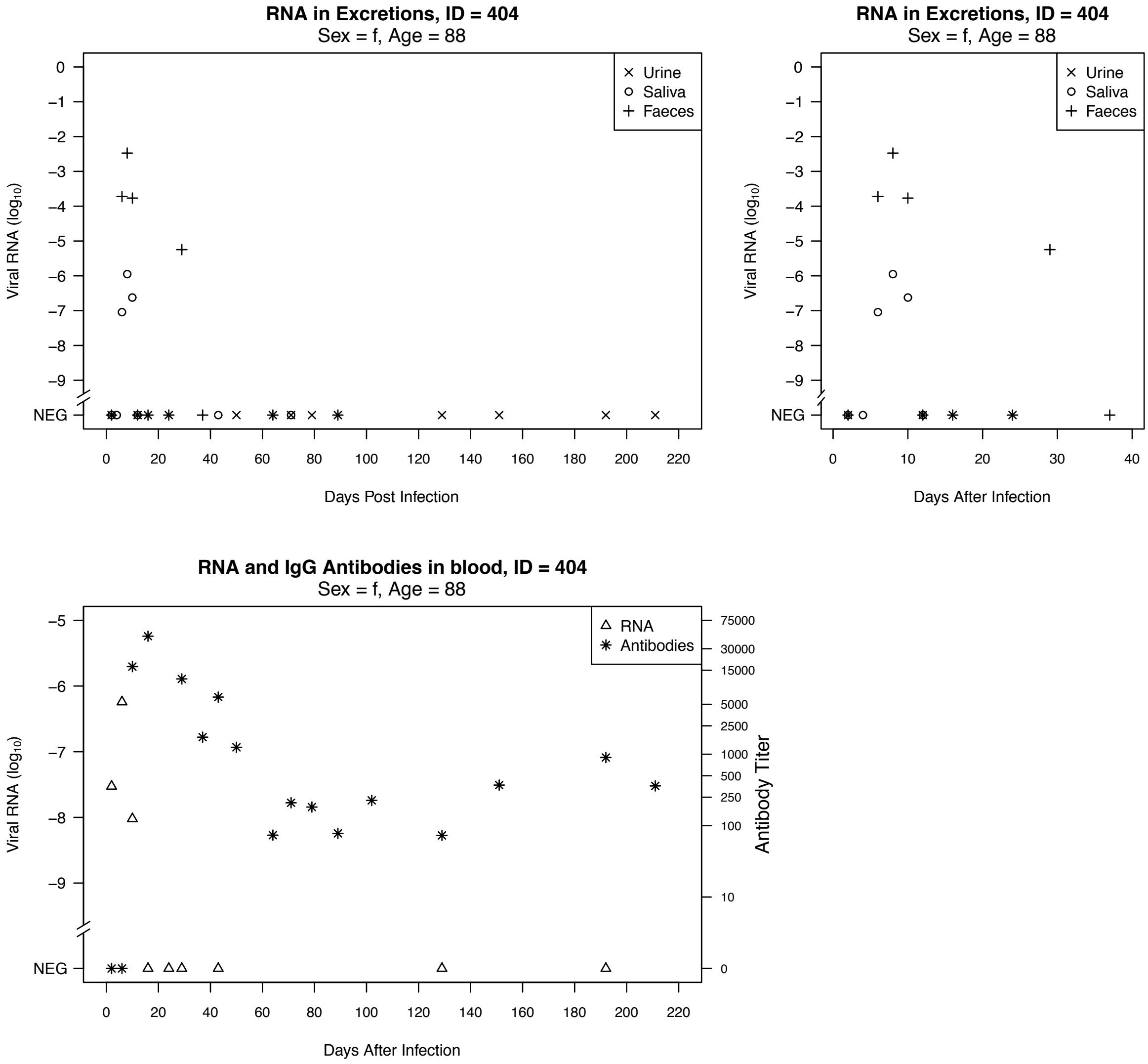

Figure 5. Antibody titers (blood) and MORV RNA (blood, excretions) for individual 4 of 15. Relative RNA levels are log-transformed ($\log_{10}$ [RNA level]) to improve plot interpretation. A value of -6 corresponds with the RNA level of a 10^{-6} dilution of the standard RNA (used for relative quantification) extracted from a 10^{6} FFU/mL virus stock, and would thus correspond with a concentration of 1 FFU/mL.

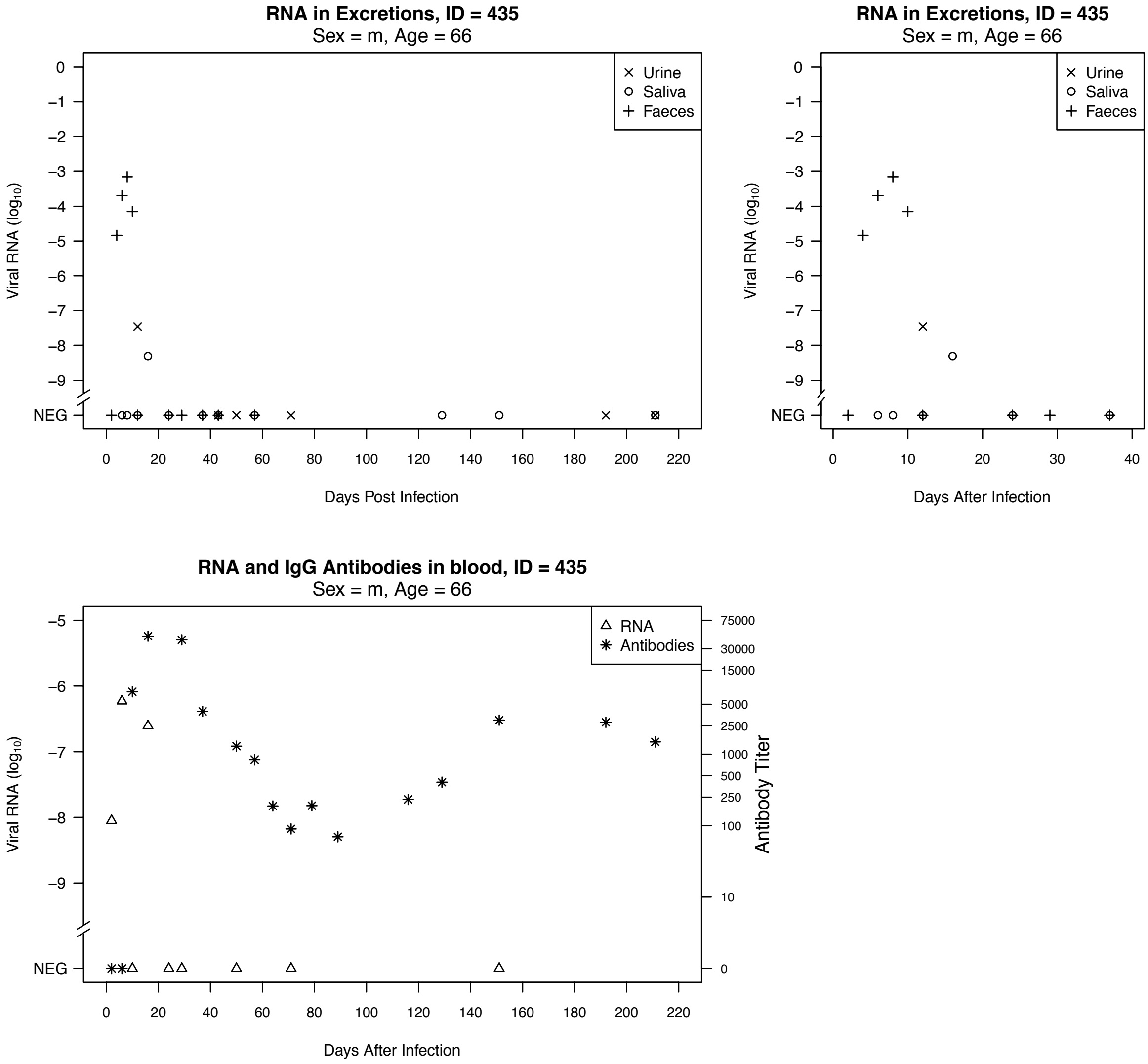

Figure 6. Antibody titers (blood) and MORV RNA (blood, excretions) for individual 5 of 15. Relative RNA levels are log-transformed ($\log_{10}$ [RNA level]) to improve plot interpretation. A value of -6 corresponds with the RNA level of a 10^{-6} dilution of the standard RNA (used for relative quantification) extracted from a 10^{6} FFU/mL virus stock, and would thus correspond with a concentration of 1 FFU/mL.

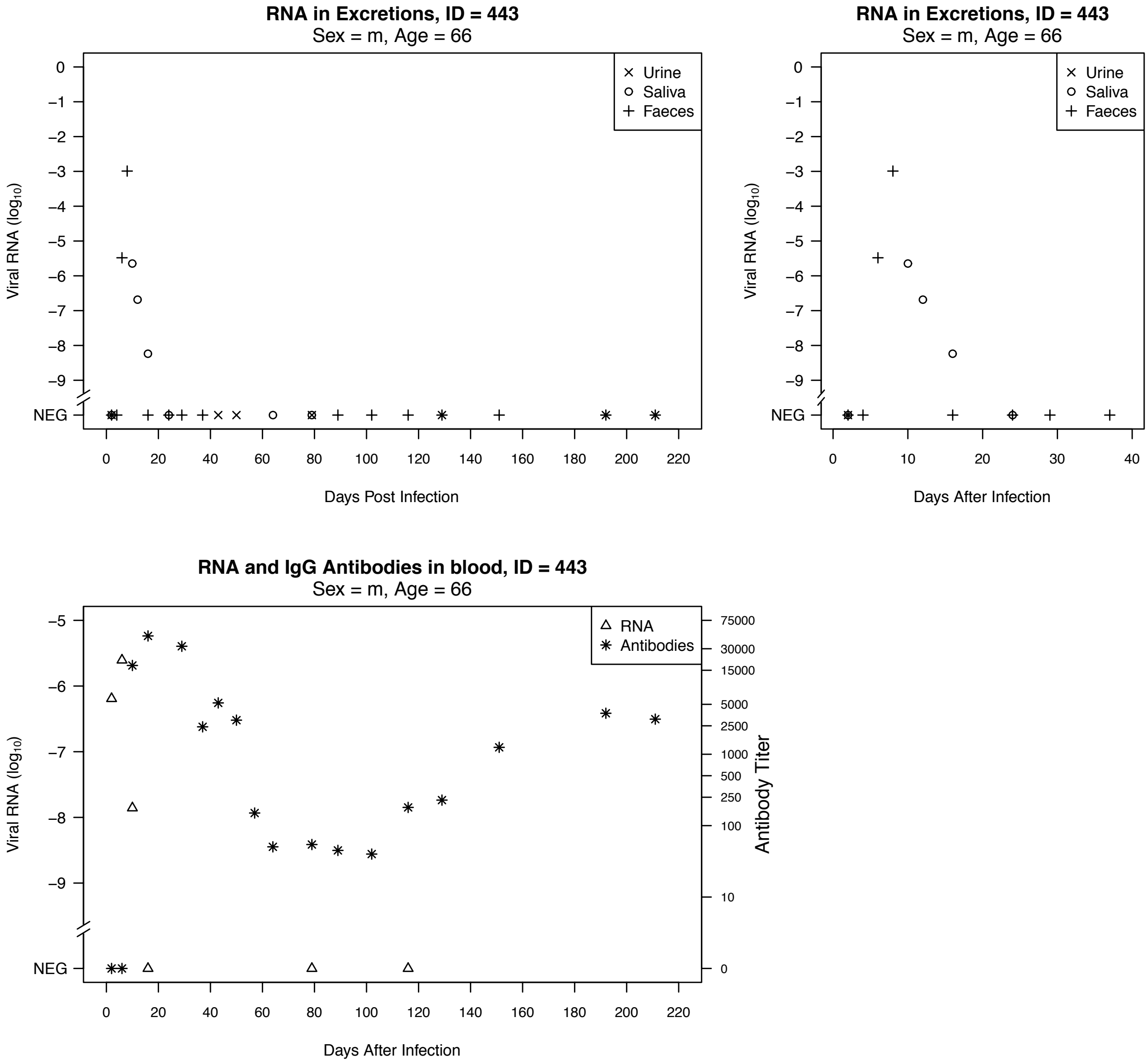

Figure 7. Antibody titers (blood) and MORV RNA (blood, excretions) for individual 6 of 15. Relative RNA levels are log-transformed ($\log_{10}$ [RNA level]) to improve plot interpretation. A value of -6 corresponds with the RNA level of a 10^{-6} dilution of the standard RNA (used for relative quantification) extracted from a 10^{6} FFU/mL virus stock, and would thus correspond with a concentration of 1 FFU/mL.

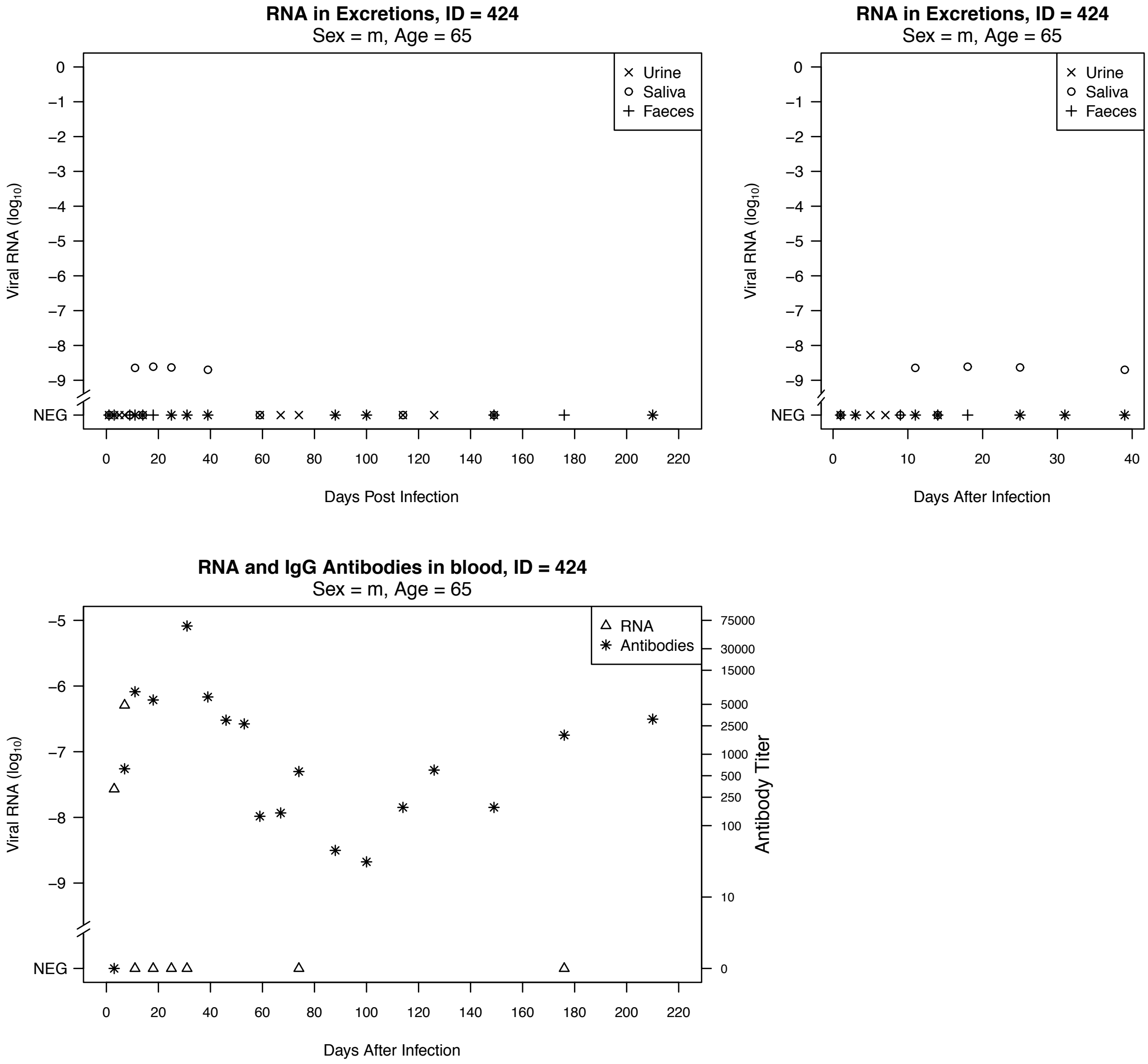

Figure 8. Antibody titers (blood) and MORV RNA (blood, excretions) for individual 7 of 15. Relative RNA levels are log-transformed ($\log_{10}$ [RNA level]) to improve plot interpretation. A value of -6 corresponds with the RNA level of a 10^{-6} dilution of the standard RNA (used for relative quantification) extracted from a 10^{6} FFU/mL virus stock, and would thus correspond with a concentration of 1 FFU/mL.

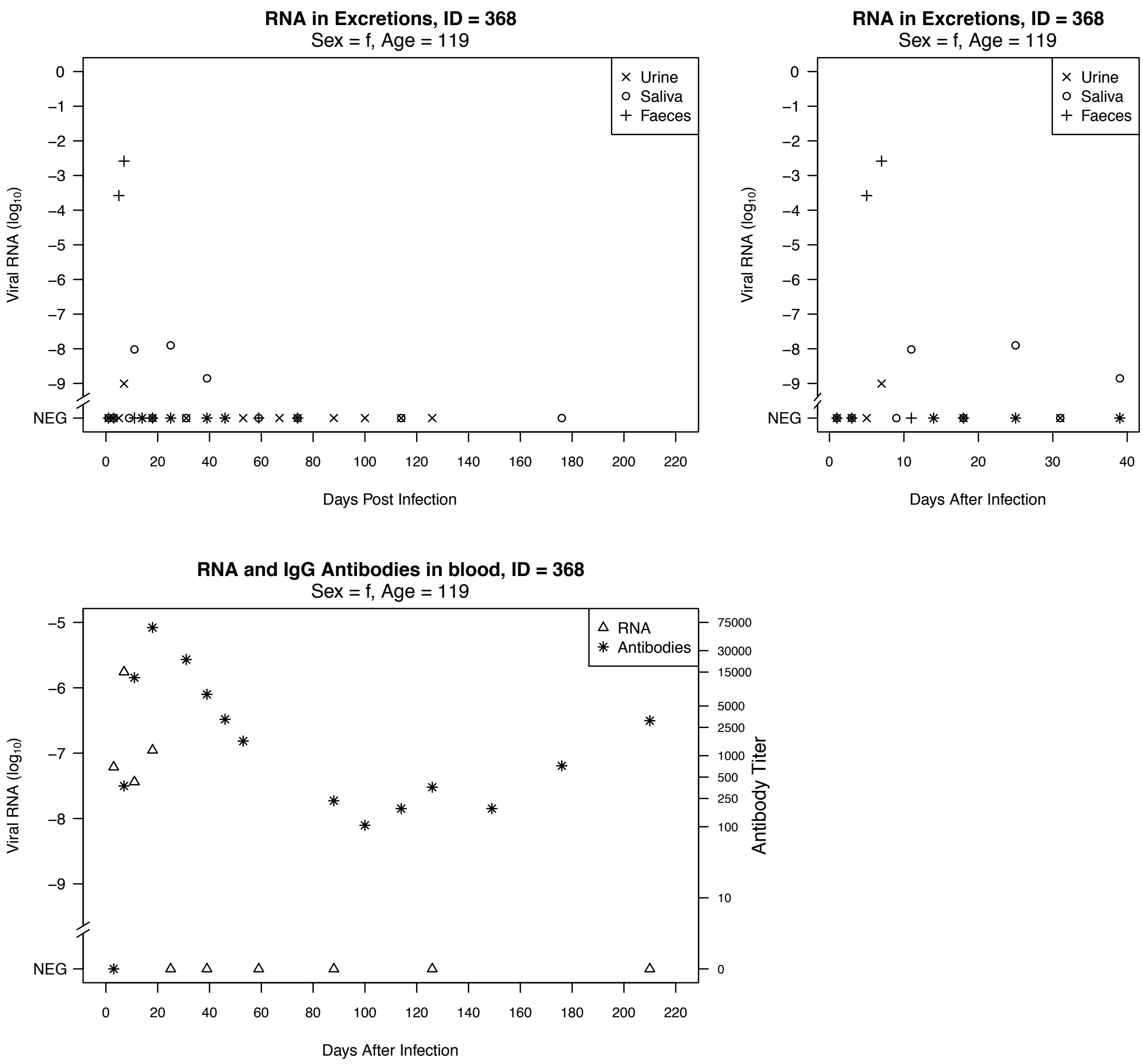

Figure 9. Antibody titers (blood) and MORV RNA (blood, excretions) for individual 8 of 15. Relative RNA levels are log-transformed ($\log_{10}$ [RNA level]) to improve plot interpretation. A value of -6 corresponds with the RNA level of a 10^{-6} dilution of the standard RNA (used for relative quantification) extracted from a 10^{6} FFU/mL virus stock, and would thus correspond with a concentration of 1 FFU/mL.

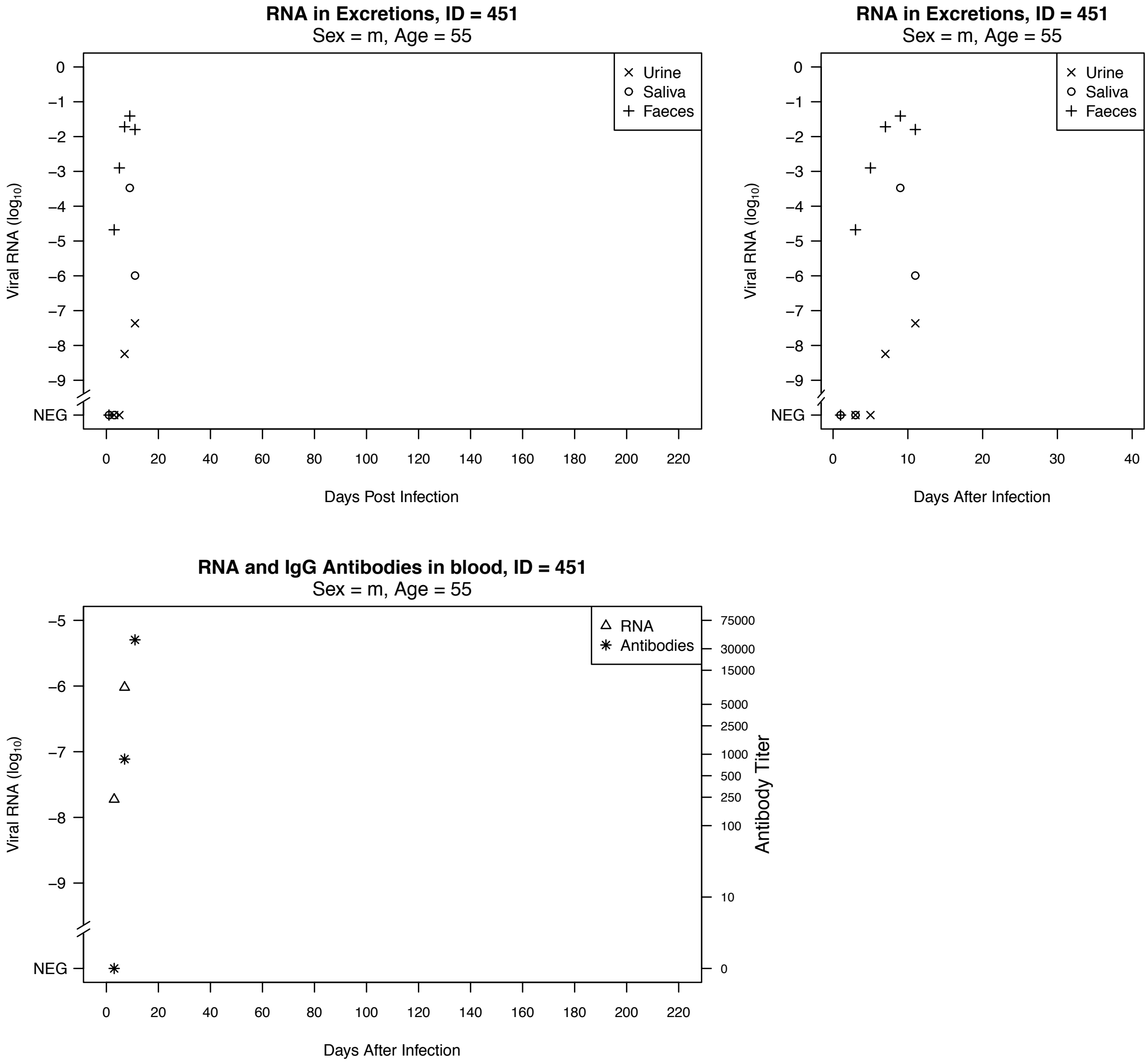

Figure 10. Antibody titers (blood) and MORV RNA (blood, excretions) for individual 9 of 15. Relative RNA levels are log-transformed ($\log_{10}$ [RNA level]) to improve plot interpretation. A value of -6 corresponds with the RNA level of a 10^{-6} dilution of the standard RNA (used for relative quantification) extracted from a 10^{6} FFU/mL virus stock, and would thus correspond with a concentration of 1 FFU/mL. This individual died on day 11 post-infection due to accidental anesthetic overdose.

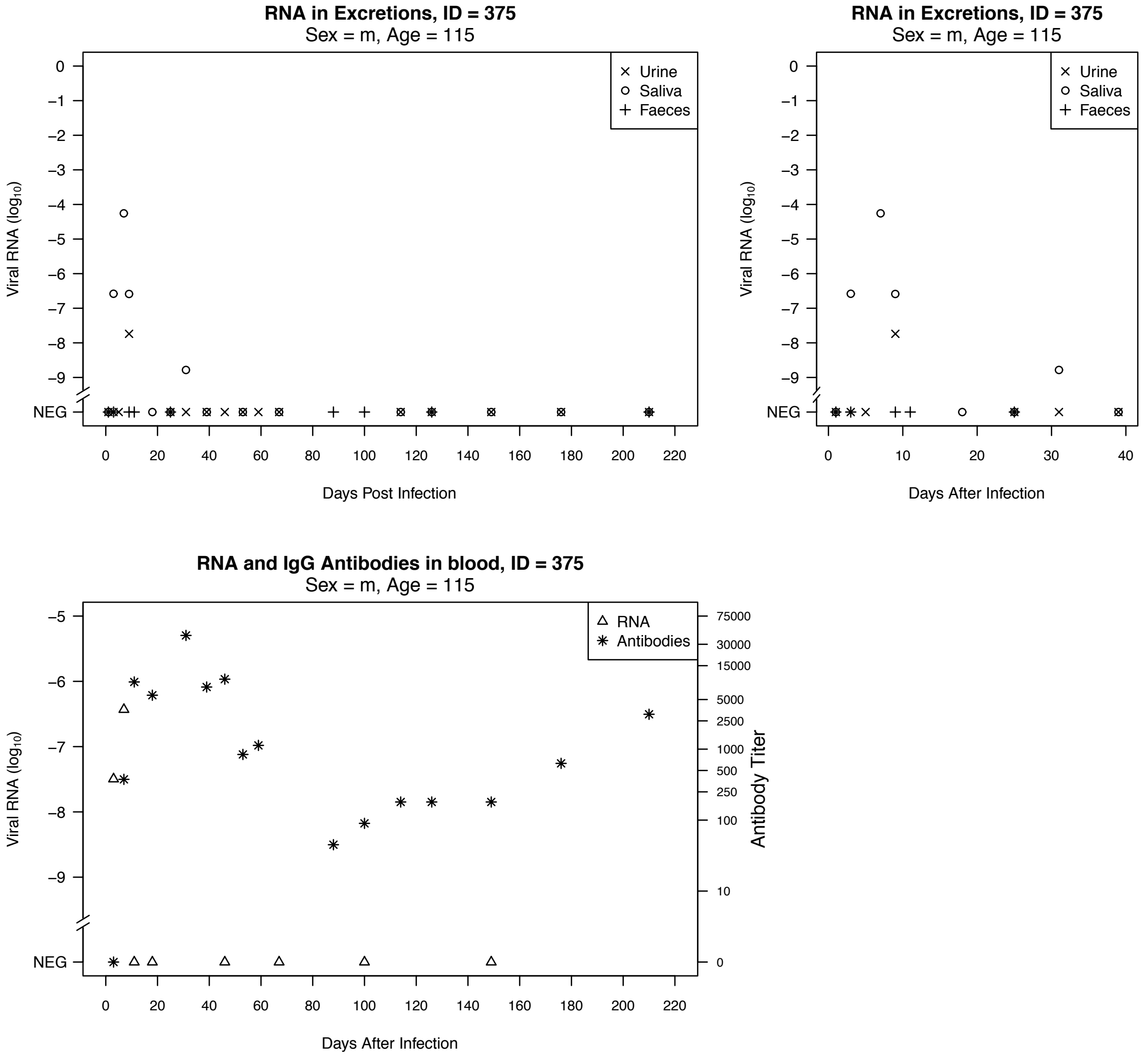

Figure 11. Antibody titers (blood) and MORV RNA (blood, excretions) for individual 10 of 15. Relative RNA levels are log-transformed ($\log_{10}$ [RNA level]) to improve plot interpretation. A value of -6 corresponds with the RNA level of a 10^{-6} dilution of the standard RNA (used for relative quantification) extracted from a 10^{6} FFU/mL virus stock, and would thus correspond with a concentration of 1 FFU/mL.

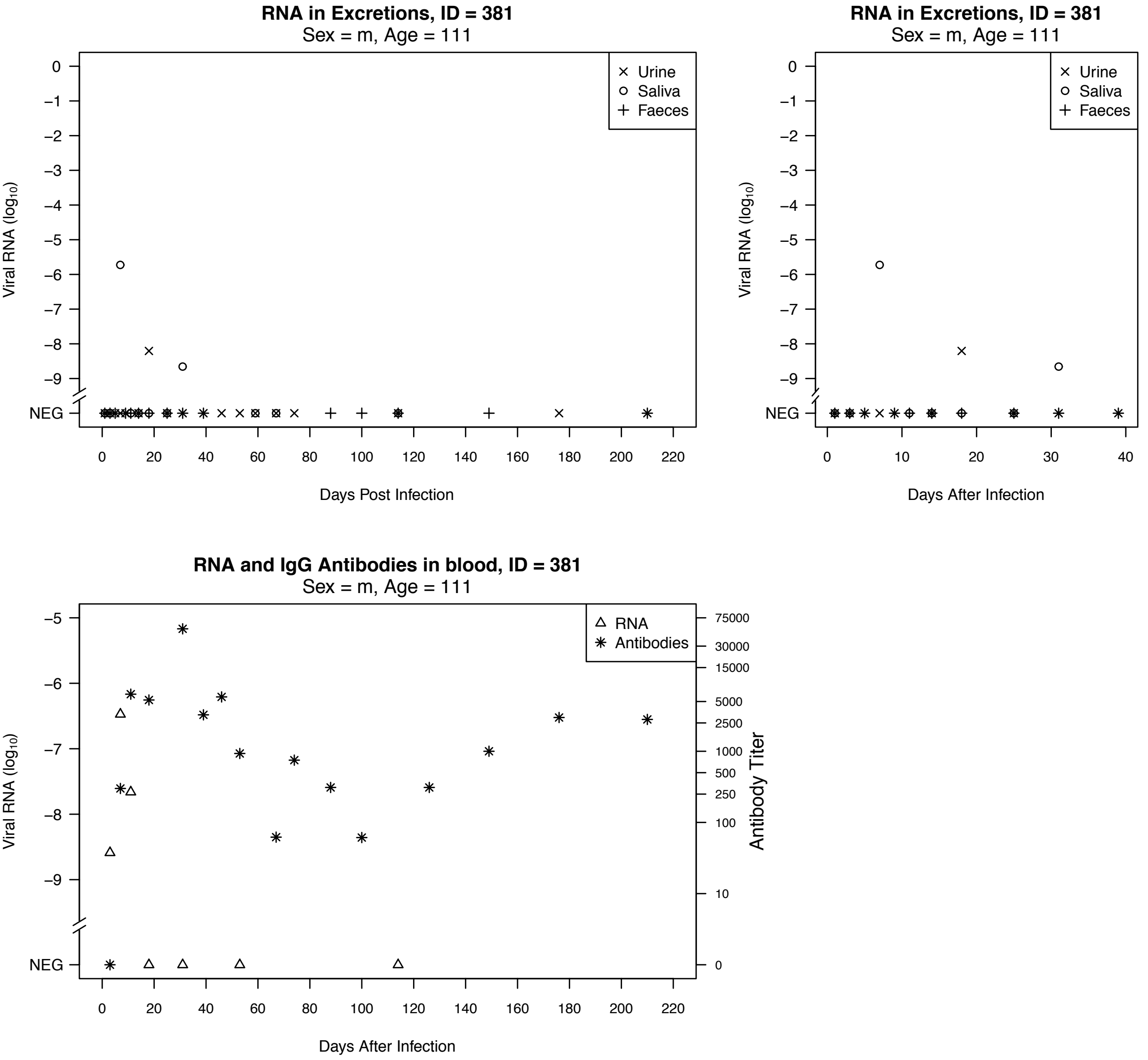

Figure 12. Antibody titers (blood) and MORV RNA (blood, excretions) for individual 11 of 15. Relative RNA levels are log-transformed ($\log_{10}$ [RNA level]) to improve plot interpretation. A value of -6 corresponds with the RNA level of a 10^{-6} dilution of the standard RNA (used for relative quantification) extracted from a 10^{6} FFU/mL virus stock, and would thus correspond with a concentration of 1 FFU/mL.

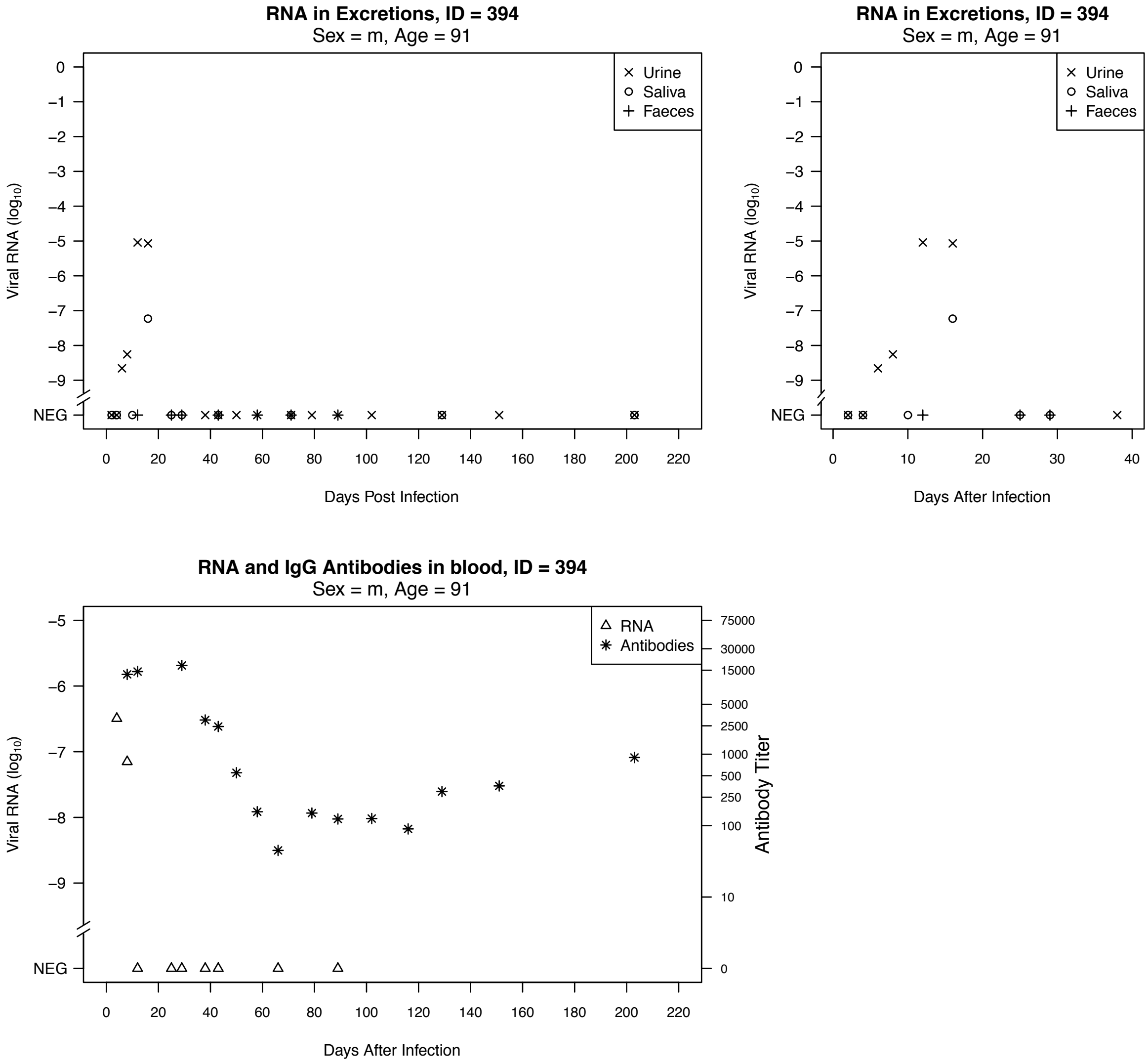

Figure 13. Antibody titers (blood) and MORV RNA (blood, excretions) for individual 12 of 15. Relative RNA levels are log-transformed ($\log_{10}$ [RNA level]) to improve plot interpretation. A value of -6 corresponds with the RNA level of a 10^{-6} dilution of the standard RNA (used for relative quantification) extracted from a 10^{6} FFU/mL virus stock, and would thus correspond with a concentration of 1 FFU/mL.

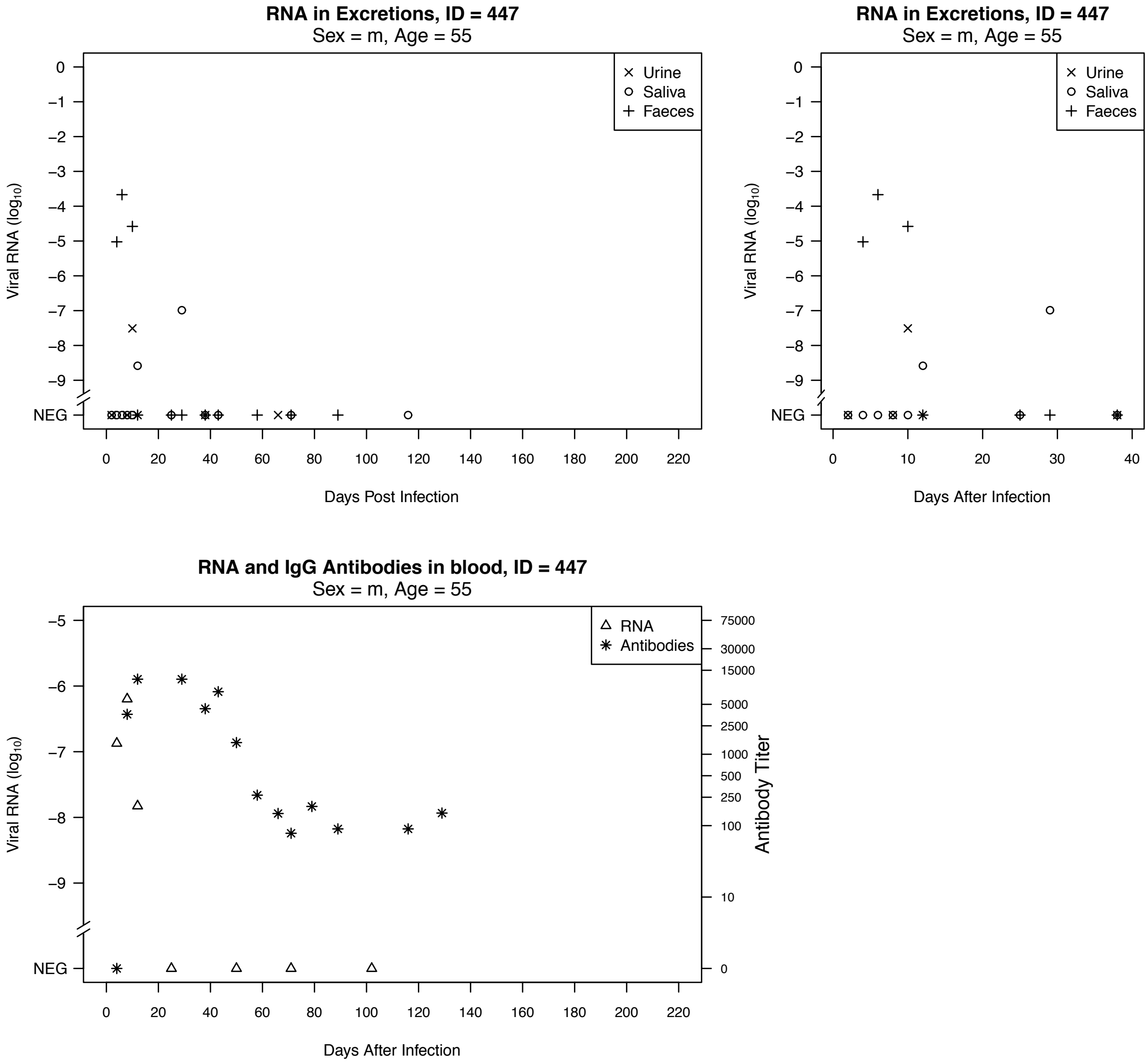

Figure 14. Antibody titers (blood) and MORV RNA (blood, excretions) for individual 13 of 15. Relative RNA levels are log-transformed ($\log_{10}$ [RNA level]) to improve plot interpretation. A value of -6 corresponds with the RNA level of a 10^{-6} dilution of the standard RNA (used for relative quantification) extracted from a 10^{6} FFU/mL virus stock, and would thus correspond with a concentration of 1 FFU/mL. This individual was euthanized on day 129 post-infection because of ulcer development caused by tail-biting.

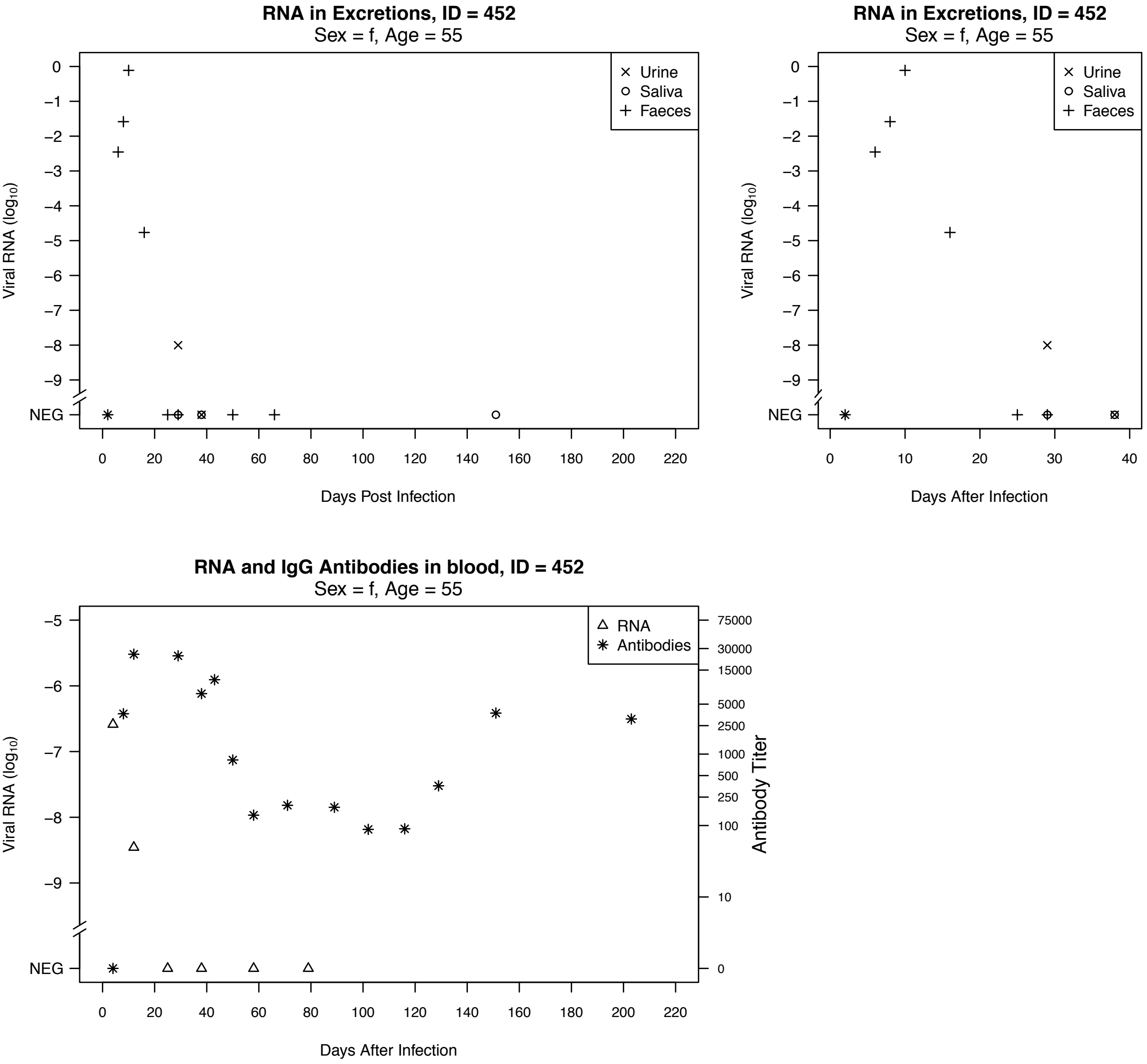

Figure 15. Antibody titers (blood) and MORV RNA (blood, excretions) for individual 14 of 15. Relative RNA levels are log-transformed ($\log_{10}$ [RNA level]) to improve plot interpretation. A value of -6 corresponds with the RNA level of a 10^{-6} dilution of the standard RNA (used for relative quantification) extracted from a 10^{6} FFU/mL virus stock, and would thus correspond with a concentration of 1 FFU/mL.

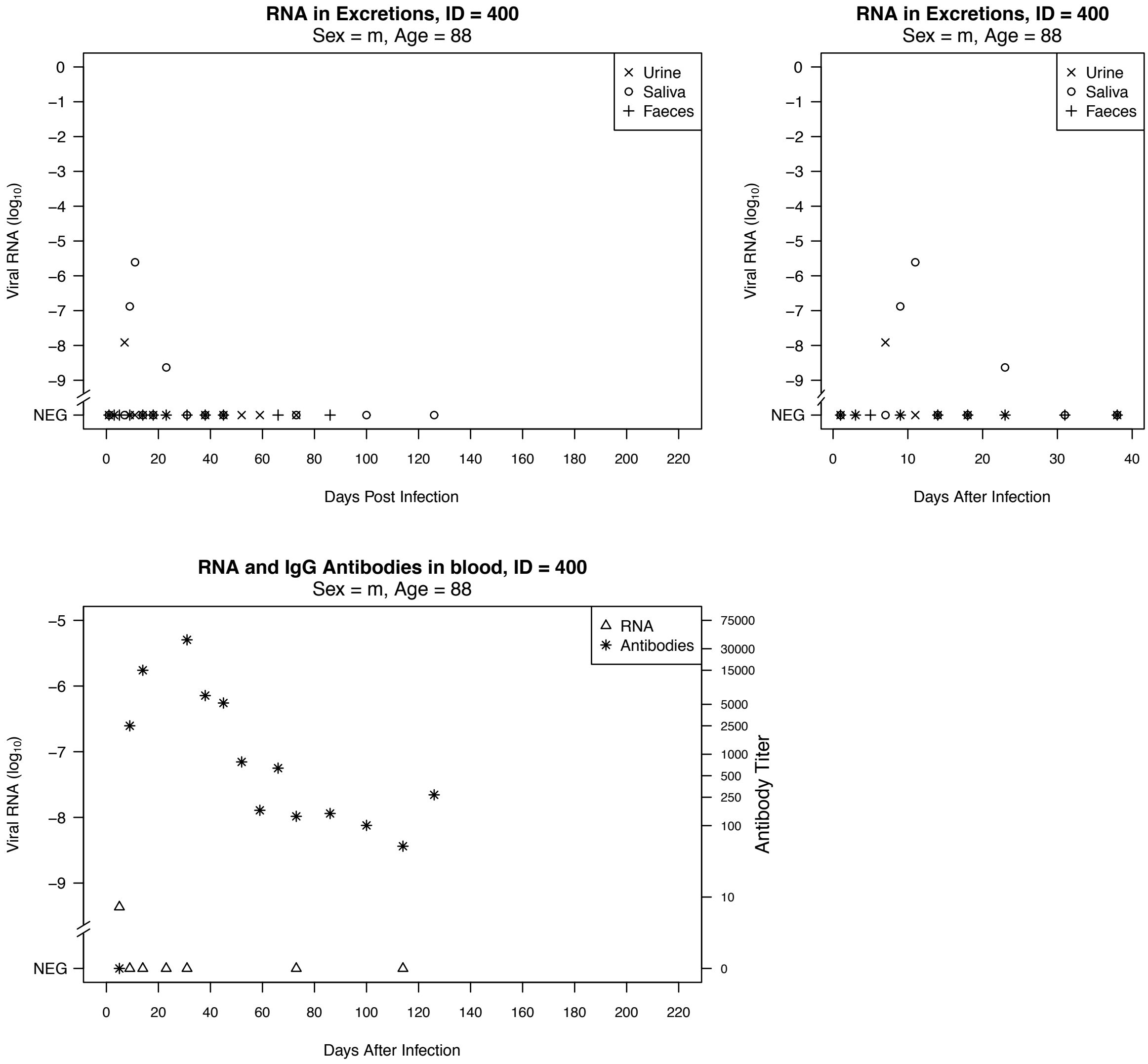

Figure 16. Antibody titers (blood) and MORV RNA (blood, excretions) for individual 15 of 15. Relative RNA levels are log-transformed ($\log_{10}$ [RNA level]) to improve plot interpretation. A value of -6 corresponds with the RNA level of a 10^{-6} dilution of the standard RNA (used for relative quantification) extracted from a 10^{6} FFU/mL virus stock, and would thus correspond with a concentration of 1 FFU/mL. This individual died naturally on day 149 post-infection.
